# Evaluation of cardiovascular toxicity of the atezolizumab and bevacizumab combination

**DOI:** 10.3389/fdsfr.2023.1213771

**Published:** 2023-08-04

**Authors:** Takahiro Niimura, Mitsuhiro Goda, Koji Miyata, Jun Matsumoto, Toshihiko Yoshioka, Hirofumi Hamano, Fuka Aizawa, Kenta Yagi, Yuki Izawa-Ishizawa, Yoshito Zamami, Keisuke Ishizawa

**Affiliations:** ^1^ Department of Clinical Pharmacology and Therapeutics, Tokushima University Graduate School of Biomedical Sciences, Tokushima, Japan; ^2^ Clinical Research Center for Developmental Therapeutics, Tokushima University Hospital, Tokushima, Japan; ^3^ Department of Pharmacy, Tokushima University Hospital, Tokushima, Japan; ^4^ Department of Personalized Medicine and Preventive Healthcare Sciences, Faculty of Medicine, Dentistry and Pharmaceutical Sciences, Okayama University, Okayama, Japan; ^5^ Department of Pharmacy, Okayama University Hospital, Okayama, Japan; ^6^ Department of General Medicine, Taoka Hospital, Tokushima, Japan

**Keywords:** atezolizumab, bevacizumab, cardiovascular toxicity, thromboembolism, adverse event

## Abstract

**Introduction:** The combination of atezolizumab, an immune checkpoint inhibitor (ICI), and bevacizumab, a vascular endothelial growth factor (VEGF) inhibitor, is the first choice for systemic therapy in hepatocellular carcinoma. Immune-related cardiovascular toxicity—myocarditis and pericarditis—are known to occur during ICI treatment. By contrast, VEGF inhibitors (VEGFIs) cause cardiovascular complications such as hypertension and heart failure. Thus, different cardiovascular toxicities have been recognized for ICIs and VEGFIs, but the impact of their combination remains unclear. Here, we aimed to investigate the cardiovascular toxicity profile of atezolizumab in combination with bevacizumab using the World Health Organization adverse event reporting database—VigiBase.

**Methods:** We analyzed data included in VigiBase till December 2022. To evaluate the frequency of reports related to atezolizumab, bevacizumab, and their combinations for 21 adverse events, we calculated the reporting odds ratio and information component. Analyses of the fatality of various cardiovascular toxicities associated with the use of each drug were performed.

**Results:** The database included 84,951, 10,595, and 2,092 reports of treatment with bevacizumab, atezolizumab, and their combination, respectively. The disproportionality signal of hypertension, arterial embolism and thrombosis, supraventricular tachyarrhythmias, heart failure, myocarditis, hemorrhage-related clinical events, venous embolism and thrombosis, cardiomyopathy, respiratory failure with combination regimen of atezolizumab and bevacizumab was detected. Signals of these adverse events were also detected treatment with either atezolizumab or bevacizumab alone. Venous embolism and thrombosis exhibited the highest fatality rate in the two drug combination (12.82%) relative to those of atezolizumab (6.19%) and bevacizumab (4.54%).

**Discussion:** Cardiovascular toxicity, owing to the combination of atezolizumab and bevacizumab, was similar to that of each single agent, and no new safety concerns were observed. Caution should be exercised when combining the two drugs since the fatality rate of thromboembolism increases with combination treatment.

## 1 Introduction

Immune checkpoint inhibitors (ICIs) exert anti-tumor effects by inducing an immune response against tumors and have become the standard treatment for several types of cancer (Motzer et al., 2022; Reck et al., 2022; Shiravand et al., 2022). Recently, combination therapy with various molecular-targeted drugs has also been investigated. For example, vascular endothelial growth factor inhibitors (VEGFIs) improve tumor microenvironment and enhance the efficacy of immunotherapy by normalizing blood vessels and promoting the infiltration and activation of immune cells, including effector T cells (Ren et al., 2021). Since the excessive production of VEGF suppresses immunity, combination therapy with ICIs and VEGFIs has been used in hepatocellular carcinoma and other types of cancer to enhance the individual anti-tumor effect of ICIs and VEGFIs (Fukumura et al., 2018).

Although the combination of VEGFIs and ICIs may enhance anti-tumor effects, both VEGFIs and ICIs are associated with cardiovascular toxicities, including hypertension, heart failure, and pericardial disease (Salem et al., 2018). Bevacizumab, a VEGFI, is associated with cardiovascular toxicities such as hypertension and thrombosis due to vascular endothelial damage caused by VEGF inhibition (Ferrara, 2004), while ICIs cause myocarditis and other conditions through activation of various immune responses, including T cells (Moslehi et al., 2021).

Therefore, VEGFIs and ICIs manifest their cardiovascular toxicity via their effects on vascular endothelial cells and immune cells, respectively. VEGF signaling affects immune cells, including T and dendritic cells, and a combination of VEGFIs and ICIs may enhance cardiovascular toxicity and increase risk (Yi et al., 2019; Ren et al., 2021). The cardiovascular toxicity profile of VEGFIs and ICIs combination has not yet been determined.

Therefore, in this study, we investigate the cardiovascular toxicity profile of atezolizumab in combination with bevacizumab using the World Health Organization (WHO) adverse event reporting database and determine the differences in the cardiovascular toxicity profiles of each drug alone.

## 2 Materials and methods

### 2.1 Database source

VigiBase, the global WHO database of adverse events, provides data on reported potential side effects of medicinal products and is maintained by the Uppsala Monitoring Centre and is the world’s largest database of adverse event reports, with more than 28 million reports collected globally since 1968 (Uppsala Monitoring Centre, UMC, 2023, VigiBase). In this study, we used data provided by VigiBase as of December 2022. VigiBase contains duplicate reports; hence, the reports determined by the Uppsala Monitoring Centre to have the potential for duplicate reporting were excluded from the analysis. Of the 33,188,305 reports, 32,520,983 were included in the analysis, excluding 667,322 reports suspected of duplication. Downloaded data were processed using SQLite databases 3.33.0 (SQLite Consortium, Charlotte, NC, United States). The requirement of informed consent from patients was waived for this study because this is an observational study using anonymized data from a global database (VigiBase), which did not involve any treatment intervention or collection of human samples.

### 2.2 Outcomes

In this study, we used the definition of adverse events as per the description provided in the Medical Dictionary for Regulatory Activities (MedDRA) developed by the International Conference on Harmonization of Technical Requirements for the Registration of Pharmaceuticals for Human Use (ICH, 2019, MedDRA). Twenty-one cardiovascular toxicities, including hypertension, hemorrhage, embolism and thrombosis, arrhythmias, and myocarditis, of atezolizumab, bevacizumab, and their combination were investigated as previously described (Salem et al., 2018; Goldman et al., 2021). Each cardiovascular toxicity is defined in Supplementary Table S1.

### 2.3 Statistical analyses

Categorical variables are summarized as frequencies and percentages. A disproportionality analysis was performed to identify the relationship between drugs and adverse events. Reporting odds ratios (RORs) and information components (IC) were used as indicators in the disproportionality analysis (Bate et al., 1998; Rothman et al., 2004; Norén et al., 2013). Adverse event reports were divided into four groups: 1) the number of reports on specific adverse events for a drug of interest, 2) the number of reports on other adverse events for a drug of interest, 3) the number of reports on specific adverse events without a drug of interest, and 4) the number of reports on other adverse events without a drug of interest. RORs were calculated as follows:

$$ROR=\left(\frac{a}{b}\right)/\left(\frac{c}{d}\right)$$

ICs were calculated using the number of expected reports no specific adverse event for the drug of interest (Nexpected) and the number of reports on a specific adverse event for the drug of interest (Nobserved), as follows:

$$IC=\log2\;\left(\left[Nobserved+0.5\right]/\;\left[Nexpected+0.5\right]\right)$$

A signal was detected when the lower limit of the 95% confidence interval (CI) for RORs and ICs exceeded 1 and 0, respectively (Bate et al., 1998; van Puijenbroek et al., 2002). For ROR, the signal was evaluated when the number of (a) was >3. For adverse events for which a signal was detected in both ROR and IC, drug-drug interaction signals were evaluated using the Ω shrinkage measure. The signal was considered detected when the lower limit of the 95% confidence interval of the Ω exceeded 0 (Norén et al., 2008; Xia et al., 2022). Analyses of the fatality of various cardiovascular toxicities associated with the use of each drug were performed. These analyses were performed using R version 4.2.1 (R Foundation for Statistical Computing, Vienna, Austria).

## 3 Results

### 3.1 Descriptive analysis

Overall, VigiBase included data from 32,520,983 reports, of which 84,951 involved bevacizumab use, 10,595 involved atezolizumab use, and 2,092 involved the use of both drugs. Approximately 60% of the reports in which age was stated reported an age of ≥45 years (23,867,872 reports stated the age vs. 14,538,974 reports stated an age of ≥45 years) (Supplementary Table S2).

The number of females in the bevacizumab group was higher than that of males (43,506 reports for females vs. 30,717 reports for males), whereas the number of males in the atezolizumab (5,478 reports for males vs. 4,194 reports for females) and two-drug combination groups (1,135 reports for males vs. 759 reports for females) was higher than that of females.

### 3.2 Disproportionality analysis

Bevacizumab treatment was associated with more reports of hypertension (95% CI min of ROR: 5.28, 95% CI min of IC: 2.31), arterial embolism and thrombosis (95% CI min of ROR: 2.26, 95% CI min of IC: 1.14), myocardial infarction (95% CI min of ROR: 1.42, 95% CI min of IC: 0.48), central nervous system ischemia (95% CI min of ROR: 2.32, 95% CI min of IC: 1.18), supraventricular tachyarrhythmias (95% CI min of ROR: 2.03, 95% CI min of IC: 0.99), ventricular tachyarrhythmias (95% CI min of ROR: 1.89, 95% CI min of IC: 0.87), heart failure (95% CI min of ROR: 1.94, 95% CI min of IC: 0.93), shock (95% CI min of ROR: 1.35, 95% CI min of IC: 0.41), pericarditis (95% CI min of ROR: 1.19, 95% CI min of IC: 0.21), hemorrhage-related clinical events (95% CI min of ROR: 2.08, 95% CI min of IC: 1), bleeding-related laboratory abnormalities (95% CI min of ROR: 1.4, 95% CI min of IC: 0.42), cerebral hemorrhage (95% CI min of ROR: 2.01, 95% CI min of IC: 0.98), pulmonary hypertension (95% CI min of ROR: 1.57, 95% CI min of IC: 0.61), venous embolism and thrombosis (95% CI min of ROR: 6.25, 95% CI min of IC: 2.57), cardiomyopathy (95% CI min of ROR: 3.68, 95% CI min of IC: 1.84), respiratory failure (95% CI min of ROR: 1.36, 95% CI min of IC: 0.42) (Table 1).

**TABLE 1:** Reporting odds ratio for cardiovascular toxicity in all reports.

Adverse event	Drug	Number of reports	ROR	95% CI min of ROR	IC	95% CI min of IC
Hypertension	Bevacizumab	5,040	5.43	5.28	2.35	2.31
Hypertension	Atezolizumab	81	0.66	0.53	−0.6	−0.97
Hypertension	Atezolizumab and bevacizumab	114	4.91	4.07	2.21	1.9
Arterial embolism and thrombosis	Bevacizumab	1,578	2.38	2.26	1.23	1.14
Arterial embolism and thrombosis	Atezolizumab	73	0.87	0.69	−0.2	−0.59
Arterial embolism and thrombosis	Atezolizumab and bevacizumab	27	1.64	1.12	0.69	0.04
Myocardial infarction	Bevacizumab	777	1.52	1.42	0.6	0.48
Myocardial infarction	Atezolizumab	65	1.01	0.79	0.02	−0.39
Myocardial infarction	Atezolizumab and bevacizumab	12	0.95	0.54	−0.07	−1.05
Central nervous system ischemia	Bevacizumab	1,276	2.46	2.32	1.28	1.18
Central nervous system ischemia	Atezolizumab	47	0.71	0.54	−0.48	−0.96
Central nervous system ischemia	Atezolizumab and bevacizumab	18	1.39	0.88	0.46	−0.33
Endocardial disorders	Bevacizumab	14	1.27	0.75	0.33	−0.57
Endocardial disorders	Atezolizumab	2	1.45	0.36	0.41	−2.18
Endocardial disorders	Atezolizumab and bevacizumab	1	3.68	0.52	0.96	−2.82
Supraventricular tachyarrhythmias	Bevacizumab	558	2.21	2.03	1.13	0.99
Supraventricular tachyarrhythmias	Atezolizumab	96	3.05	2.49	1.58	1.24
Supraventricular tachyarrhythmias	Atezolizumab and bevacizumab	13	2.08	1.21	1	0.06
Ventricular tachyarrhythmias	Bevacizumab	169	2.2	1.89	1.13	0.87
Ventricular tachyarrhythmias	Atezolizumab	11	1.14	0.63	0.19	−0.84
Ventricular tachyarrhythmias	Atezolizumab and bevacizumab	0	0	0	−2.26	−12.59
Heart failure	Bevacizumab	829	2.07	1.94	1.04	0.93
Heart failure	Atezolizumab	81	1.62	1.3	0.68	0.32
Heart failure	Atezolizumab and bevacizumab	18	1.82	1.15	0.83	0.04
Shock	Bevacizumab	606	1.46	1.35	0.54	0.41
Shock	Atezolizumab	54	1.04	0.8	0.06	−0.4
Shock	Atezolizumab and bevacizumab	5	0.49	0.2	−0.97	−2.53
Myocarditis	Bevacizumab	13	0.14	0.08	−2.83	−3.77
Myocarditis	Atezolizumab	73	6.18	4.91	2.57	2.18
Myocarditis	Atezolizumab and bevacizumab	14	5.99	3.54	2.35	1.44
Pericarditis	Bevacizumab	171	1.39	1.19	0.47	0.21
Pericarditis	Atezolizumab	63	4.11	3.21	2	1.58
Pericarditis	Atezolizumab and bevacizumab	6	1.98	0.89	0.88	−0.54
Hemorrhage-related clinical events	Bevacizumab	5,587	2.14	2.08	1.04	1
Hemorrhage-related clinical events	Atezolizumab	206	0.6	0.52	−0.71	−0.94
Hemorrhage-related clinical events	Atezolizumab and bevacizumab	96	1.46	1.19	0.52	0.18
Bleeding-related laboratory abnormalities	Bevacizumab	75	1.76	1.4	0.8	0.42
Bleeding-related laboratory abnormalities	Atezolizumab	1	0.19	0.03	−1.96	−5.74
Bleeding-related laboratory abnormalities	Atezolizumab and bevacizumab	1	0.95	0.13	−0.05	−3.83
Cerebral hemorrhage	Bevacizumab	1,133	2.13	2.01	1.08	0.98
Cerebral hemorrhage	Atezolizumab	52	0.78	0.59	−0.36	−0.82
Cerebral hemorrhage	Atezolizumab and bevacizumab	12	0.91	0.51	−0.13	−1.11
Pulmonary hypertension	Bevacizumab	163	1.84	1.57	0.87	0.61
Pulmonary hypertension	Atezolizumab	6	0.54	0.24	−0.84	−2.25
Pulmonary hypertension	Atezolizumab and bevacizumab	0	0	0	−2.43	−12.75
Vasculitis	Bevacizumab	94	0.95	0.78	−0.07	−0.41
Vasculitis	Atezolizumab	18	1.47	0.92	0.53	−0.26
Vasculitis	Atezolizumab and bevacizumab	2	0.82	0.21	−0.23	−2.82
Temporal arteritis/polymyalgia rheumatica	Bevacizumab	10	0.62	0.33	−0.67	−1.75
Temporal arteritis/polymyalgia rheumatica	Atezolizumab	6	2.96	1.33	1.36	−0.05
Temporal arteritis/polymyalgia rheumatica	Atezolizumab and bevacizumab	0	0	0	−0.85	−11.17
Venous embolism and thrombosis	Bevacizumab	2,774	6.49	6.25	2.64	2.57
Venous embolism and thrombosis	Atezolizumab	113	2.05	1.7	1.02	0.71
Venous embolism and thrombosis	Atezolizumab and bevacizumab	39	3.6	2.63	1.78	1.25
Bradyarrhythmias	Bevacizumab	120	1.08	0.9	0.11	−0.19
Bradyarrhythmias	Atezolizumab	13	0.94	0.55	−0.09	−1.03
Bradyarrhythmias	Atezolizumab and bevacizumab	4	1.46	0.55	0.48	−1.29
Cardiomyopathy	Bevacizumab	365	4.08	3.68	2.01	1.84
Cardiomyopathy	Atezolizumab	33	2.94	2.09	1.51	0.93
Cardiomyopathy	Atezolizumab and bevacizumab	11	4.97	2.75	2.08	1.05
Respiratory failure	Bevacizumab	583	1.48	1.36	0.56	0.42
Respiratory failure	Atezolizumab	113	2.31	1.92	1.19	0.88
Respiratory failure	Atezolizumab and bevacizumab	19	1.96	1.25	0.93	0.16

CI, confidence interval; ROR, reporting odds ratios; IC, information components.

In atezolizumab users, a significant increase in the reports of supraventricular tachyarrhythmias (95% CI min of ROR: 2.49, 95% CI min of IC: 1.24), heart failure (95% CI min of ROR: 1.3, 95% CI min of IC: 0.32), myocarditis (95% CI min of ROR: 4.91, 95% CI min of IC: 2.18), pericarditis (95% CI min of ROR: 3.21, 95% CI min of IC: 1.58), venous embolism and thrombosis (95% CI min of ROR: 1.7, 95% CI min of IC: 0.71), cardiomyopathy (95% CI min of ROR: 2.09, 95% CI min of IC: 0.93), respiratory failure (95% CI min of ROR: 1.92, 95% CI min of IC: 0.88) was observed.

The disproportionality signal of hypertension (95% CI min of ROR: 4.07, 95% CI min of IC: 1.9), arterial embolism and thrombosis (95% CI min of ROR: 1.12, 95% CI min of IC: 0.04), supraventricular tachyarrhythmias (95% CI min of ROR: 1.21, 95% CI min of IC: 0.06), heart failure (95% CI min of ROR: 1.15, 95% CI min of IC: 0.04), myocarditis (95% CI min of ROR: 3.54, 95% CI min of IC: 1.44), hemorrhage-related clinical events (95% CI min of ROR: 1.19, 95% CI min of IC: 0.18), venous embolism and thrombosis (95% CI min of ROR: 2.63, 95% CI min of IC: 1.25), cardiomyopathy (95% CI min of ROR: 2.75, 95% CI min of IC: 1.05), respiratory failure (95% CI min of ROR: 1.25, 95% CI min of IC: 0.16) with the two-drug combination regimen of bevacizumab and atezolizumab was detected.

A disproportionality analysis by sex and age was also performed (Supplementary Tables S3–S6). Arterial embolism and thrombosis (male[95% CI min of ROR: 0.49, 95% CI min of IC: −1.19], female[95% CI min of ROR: 1.87, 95% CI min of IC: 0.65]), heart Failure (male[95% CI min of ROR: 0.73, 95% CI min of IC: −0.68], female[95% CI min of ROR: 1.56, 95% CI min of IC: 0.29]), respiratory failure (male[95% CI min of ROR: 0.72, 95% CI min of IC: −0.7], female[95% CI min of ROR: 1.79, 95% CI min of IC: 0.5]), myocarditis (male[95% CI min of ROR: 2.35, 95% CI min of IC: 0.83], female[95% CI min of ROR: 1.06, 95% CI min of IC: −1.23]), hemorrhage-related clinical events (male[95% CI min of ROR: 1.17, 95% CI min of IC: 0.13], female[95% CI min of ROR: 0.86, 95% CI min of IC: −0.32]), cardiomyopathy (male[95% CI min of ROR: 1.77, 95% CI min of IC: 0.27], female[95% CI min of ROR: 1.5, 95% CI min of IC: −0.46])showed differences in the frequency of reports in the stratified analysis by sex for atezolizumab and bevacizumab users.

In the age-stratified analysis, differences were observed in the frequency of event reports for heart failure (young[95% CI min of ROR: 1.82, 95% CI min of IC: 0.63], old[95% CI min of ROR: 0.17, 95% CI min of IC: −3.02]), hemorrhage-related clinical events (young[95% CI min of ROR: 1.14, 95% CI min of IC: 0.11], old[95% CI min of ROR: 0.58, 95% CI min of IC: −0.89]), cardiomyopathy (young[95% CI min of ROR: 2.97, 95% CI min of IC: 0.99], old[95% CI min of ROR: 0.46, 95% CI min of IC: −2.88]), respiratory failure (young[95% CI min of ROR: 1.18, 95% CI min of IC: 0.03], old[95% CI min of ROR: 1.05, 95% CI min of IC: −0.42]) for atezolizumab and bevacizumab users.

For adverse events in which both ROR and IC signals were detected, drug-drug interactions were evaluated using the Ω shrinkage measure (Table 2). Five adverse events were evaluated, namely, supraventricular tachyarrhythmias (Ω: −0.99, 95% CI lower limit: −1.78), heart failure (Ω: −0.54, 95% CI lower limit: −1.21), venous embolism and thrombosis (Ω: −1.01, 95% CI lower limit: −1.47), cardiomyopathy (Ω: −0.26, 95% CI lower limit: −1.11), and respiratory failure (Ω: −0.49, 95% CI lower limit: −1.14). No signal was detected in any of the adverse events.

**TABLE 2:** Drug-drug interaction signals for cardiovascular toxicity in all reports.

Drug 1	Drug 2	Adverse event	Ω	95% CI min of Ω
Bevacizumab	Atezolizumab	Supraventricular tachyarrhythmias	−0.99	−1.78
Bevacizumab	Atezolizumab	Heart failure	−0.54	−1.21
Bevacizumab	Atezolizumab	Venous embolism and thrombosis	−1.01	−1.47
Bevacizumab	Atezolizumab	Cardiomyopathy	−0.26	−1.11
Bevacizumab	Atezolizumab	Respiratory failure	−0.49	−1.14

CI, confidence interval.

### 3.3 Fatality rate analysis

**FIGURE 1:**
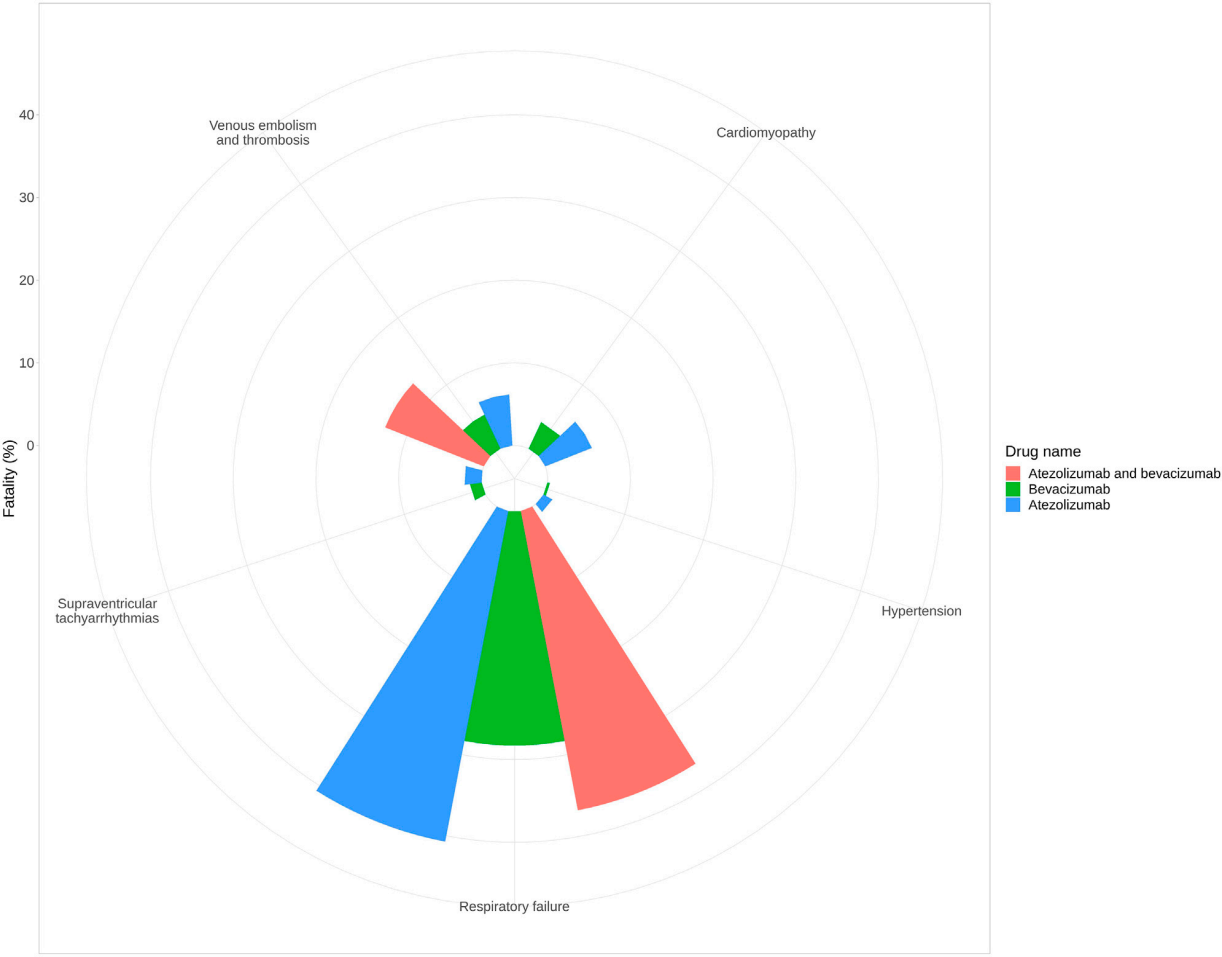
Fatality rate of each adverse event.

Fatality rates were evaluated for five adverse events in which signals were detected for the combination of atezolizumab and bevacizumab (Figure 1). Venous embolism and thrombosis exhibited the highest fatality rate in the two-drug combination (12.82%) compared with that of atezolizumab (6.19%) or bevacizumab (4.54%) alone. In the case of respiratory failure, the fatality rate of two-drug combination (36.84%) was lower than that of atezolizumab (40.71%) but higher than that of bevacizumab (28.30%). No deaths were reported due to cardiomyopathy (atezolizumab: 6.06%, bevacizumab: 3.56%), hypertension (atezolizumab: 1.23%, bevacizumab: 0.32%), or supraventricular tachyarrhythmias (atezolizumab: 2.08%, bevacizumab: 1.43%) for the two-drug combination group.

## 4 Discussion

This study systematically analyzed the world’s largest adverse events database—VigiBase—to determine the cardiovascular toxicity profile of atezolizumab, bevacizumab, and their combination. The cardiovascular toxicity of the combination of atezolizumab and bevacizumab can be predicted from the cardiovascular toxicities of each drug, and no synergistic increase in adverse events was observed when the two drugs were co-administered. With regard to thromboembolism, a trend toward a higher fatality rate was demonstrated for the combination of atezolizumab and bevacizumab relative to their individual use.

In addition to the combination of atezolizumab and bevacizumab for hepatocellular carcinoma treatment, ICI- and VEGFI-based anticancer therapies are being developed for various cancers, such as gastric and gastroesophageal adenocarcinomas (Saeed et al., 2021). Particularly, a VEGF inhibitor in combination with a PD-1/PD-L1 inhibitor has shown favorable results in PD-1/PD-L1 inhibitor-resistant patients, and the combination therapy of ramucirumab and pembrolizumab is under investigation (Reckamp et al., 2022). However, there is limited data on the cardiovascular toxicity profile of the two types of inhibitors when used together.

To our knowledge, this is the largest study to investigate the cardiovascular toxicity profile of atezolizumab in combination with bevacizumab. We utilized the global WHO database of spontaneous adverse event reports to investigate a wide range of cardiovascular toxicities that are difficult to assess in individual clinical trials. The results of this study suggest that management should consider the respective cardiovascular toxicity profiles when both drugs are used in combination.

In the current study, we observed a trend toward a higher lethality rate of venous embolism and thrombosis when ICIs and VEGFIs were used together compared with their individual use. The mechanisms involved in the increased frequency of thromboembolism with ICIs remain unclear but include increased tissue factor production in monocytes associated with T-cell activation (Zou et al., 2021). In addition, a higher risk of venous thromboembolism has been reported in patients with c-reactive protein flares early after ICI administration (Moik et al., 2022), and increased secretion of various cytokines associated with inflammatory responses may contribute to coagulation abnormalities. The possible mechanisms of thromboembolism caused by VEGFIs include exposure to procoagulant phospholipids under the endothelium by inhibiting endothelial cell regeneration and decreased production of nitric oxide and prostacyclin (Kamba and McDonald, 2007; Nalluri et al., 2008). Therefore, it is possible that a potent reaction occurs when the two drugs were used together since each has a different mechanism contributing to thromboembolism formation. More detailed studies will be needed in the future regarding the risk of thromboembolism when both drugs are used together.

Thromboembolism is a multifactorial condition with various risk factors (Eichinger, 2016; Hamza and Mousa, 2020). Studies evaluating the risk of thromboembolism associated with cancer chemotherapy suggest that the risk of thromboembolism is significantly higher in patients older than 70 years than that in those younger (Vergati et al., 2013). Regarding sex, females are reportedly at risk for cancer-related thromboembolism, including patients not receiving chemotherapy (Khorana et al., 2007). Similar to the results of studies on these risk factors, the present study showed that thromboembolism was reported more frequently in elderly patients and females among those who received atezolizumab plus bevacizumab. By contrast, thromboembolism was reported less frequently in males who received atezolizumab plus bevacizumab than in patients who did not. Since the incidence of thromboembolism is influenced by the type of cancer and the presence or absence of radiation therapy, more detailed studies on the effect of sex differences on the incidence of thromboembolism associated with atezolizumab plus bevacizumab are needed (Abdol Razak et al., 2018).

This study has some limitations. First, this study was conducted using a database of spontaneous adverse event reports, and it was not possible to determine the causal relationship between each drug and the reported adverse events. The study only reports an association between the drugs and the adverse events, and more prospective observational studies should be conducted in the future. However, the results of previous studies were similar with regard to the risk of cardiovascular toxicity when atezolizumab and bevacizumab were used individually (Economopoulou et al., 2015; Totzeck et al., 2017; Ball et al., 2019; Gan et al., 2022). Second, the past treatment and medical history of the patients included in this study are unclear. Various anticancer drugs and underlying diseases act as risk factors for cardiovascular diseases. There may be differences in the presence or absence of risk factors between the use and non-use groups for each drug. Third, there is a possibility of adverse event reporting bias since VigiBase accumulates data from various sources, including medical professionals and pharmaceutical companies. In addition, management practices for adverse events may have changed depending on the reporting year.

To the best of our knowledge, no other studies have investigated the cardiovascular toxicity profile of ICIs in combination with anti-VEGF antibodies. Antibodies have different targets of action and, therefore, different adverse events. In the current study, we found that the adverse event profiles of both drugs, when used in combination, reflect the adverse event profiles of each drug when used alone, but with some differences. Cardiovascular toxicity induced by these drugs can sometimes be lethal and requires close monitoring. The results of this study contribute to the management strategies necessary to ensure the safety of cancer immunotherapy.

## Data Availability

The data analyzed in this study is subject to the following licenses/restrictions: Data will be made available upon requests directed to the corresponding author and after approval of a proposal. Requests to access these datasets should be directed to TN, niimura@tokushima-u.ac.jp.
