# Supplementary material for: Evaluation of cardiovascular toxicity of the atezolizumab and bevacizumab combination
Source: Front Drug Saf Regul. 2023 Aug 4;3:1213771. doi: 10.3389/fdsfr.2023.1213771 (PMC12443078; doi:10.3389/fdsfr.2023.1213771)
Supplement: Supplementary file 1 [file Table1.DOCX]

Supplementary Material

Evaluation of cardiovascular toxicity of the atezolizumab and bevacizumab combination

Takahiro Niimura, Mitsuhiro Goda^*^, Koji Miyata, Jun Matsumoto, Hirofumi Hamano, Fuka Aizawa, Kenta Yagi, Yuki Izawa-Ishizawa, Yoshito Zamami, Keisuke Ishizawa

*** Correspondence:** Mitsuhiro Goda: mgoda@tokushima-u.ac.jp

# Supplementary Tables

**Supplementary Table 1**. Definition of outcomes

| **Adverse event groups** | **MedDRA Terms** |
| --- | --- |
| Hypertension | Hypertension (SMQ narrow 25.0) |
| Arterial embolism and thrombosis | Embolic and thrombotic events, arterial (SMQ narrow 25.0) |
| Myocardial infarction | Myocardial infarction (SMQ narrow 25.0) |
| Central nervous system ischemia | Ischemic central nervous system vascular conditions (SMQ narrow  25.0) |
| Bradyarrhythmias | Bradyarrhythmia terms, nonspecific (SMQ narrow 25.0)  Conduction defects (SMQ narrow 25.0)  Disorders of sinus node function (SMQ narrow 25.0) |
| Endocardial disorders | Endocardial disorders (HLGT 25.0) |
| Supraventricular tachyarrhythmias | Supraventricular tachyarrhythmias (SMQ narrow 25.0) |
| Ventricular tachyarrhythmias | Ventricular tachyarrhythmias (SMQ narrow 25.0) |
| Cardiomyopathy | Cardiomyopathy (SMQ narrow 25.0) |
| Respiratory failure | Respiratory failure (SMQ narrow 25.0) |
| Heart failure | Cardiac failure (SMQ narrow 25.0) |
| Shock | Shock-associated circulatory or cardiac conditions (excl torsade de pointes) (SMQ narrow 25.0) |
| Myocarditis | Non-infectious myocarditis (HLT 25.0) |
| Pericarditis | Non-infectious pericarditis (HLT 25.0) and pericardial disorders NEC (HLT 25.0) |
| Venous embolism and thrombosis | Embolic and thrombotic events, venous (SMQ narrow 25.0) |
| Hemorrhage (clinical events) | Hemorrhage terms (excl laboratory terms) (SMQ narrow 25.0) |
| Bleeding-related laboratory abnormalities | Hemorrhage laboratory terms (SMQ narrow 25.0) |
| Cerebral hemorrhage | Hemorrhagic central nervous system vascular conditions (SMQ narrow 25.0) |
| Pulmonary hypertension | Pulmonary hypertension (SMQ narrow 25.0) |
| Vasculitis | Vasculitis (SMQ narrow 25.0) |
| Temporal arteritis/Polymyalgia rheumatica | Polymyalgia rheumatica (PT 25.0) and temporal arteritis (PT 25.0) |

MedDRA: Medical Dictionary for Regulatory Activities; SMQ: standardized MedDRA query; HLGT: high-level group term; HLT: high-level term; PT: preferred term

**Supplementary Table 2**. Characteristics of users in each drug

|  | All | Bevacizumab user | Atezolizumab user | Bevacizumab and atezolizumab user |
| --- | --- | --- | --- | --- |
|  | (32,520,983) | (84,951) | (10,595) | (2,092) |
| Age |  |  |  |  |
| 0–27 days | 50,105 | 10 | 1 | NA |
| 28 days to 23 months | 648,950 | 81 | 3 | NA |
| 2–11 years | 904,181 | 339 | 5 | NA |
| 12–17 years | 715,818 | 216 | 4 | 1 |
| 18–44 years | 7,009,844 | 5,421 | 448 | 94 |
| 45–64 years | 7,972,870 | 25,740 | 3,136 | 651 |
| 65–74 years | 3,708,066 | 16,234 | 2,886 | 580 |
| ≥75 years | 2,858,038 | 7,637 | 1,444 | 299 |
| Unknown | 8,653,111 | 29,273 | 2,668 | 467 |
| Sex |  |  |  |  |
| Female | 18,651,597 | 43,506 | 4,194 | 759 |
| Male | 11,961,885 | 30,717 | 5,478 | 1,135 |
| Unknown | 1,907,501 | 10,728 | 923 | 198 |
| Region |  |  |  |  |
| Africa | 297,198 | 216 | 8 | 3 |
| Region of the Americas | 15,920,701 | 42,166 | 3,805 | 727 |
| South-east Asia | 1,104,440 | 966 | 918 | 88 |
| European | 8,662,203 | 25,420 | 4,369 | 964 |
| Eastern Mediterranean | 525,097 | 525 | 158 | 28 |
| Western Pacific | 6,011,344 | 15,658 | 1,337 | 282 |
| Report year |  |  |  |  |
| 2000 | 369,099 | 15 | NA | NA |
| 2001 | 315,796 | 3 | NA | NA |
| 2002 | 146,423 | 9 | NA | NA |
| 2003 | 177,619 | 4 | NA | NA |
| 2004 | 218,314 | 15 | NA | NA |
| 2005 | 344,251 | 812 | NA | NA |
| 2006 | 289,133 | 1,131 | NA | NA |
| 2007 | 32,319 | 100 | NA | NA |
| 2008 | 617,955 | 3,598 | NA | NA |
| 2009 | 514,105 | 2,603 | NA | NA |
| 2010 | 831,844 | 2,880 | NA | NA |
| 2011 | 1,087,163 | 3,401 | NA | NA |
| 2012 | 842,240 | 4,057 | 1 | NA |
| 2013 | 648,902 | 7,973 | NA | NA |
| 2014 | 1,740,216 | 7,537 | 4 | NA |
| 2015 | 2,037,553 | 6,659 | 25 | 6 |
| 2016 | 1,788,005 | 6,153 | 60 | 18 |
| 2017 | 2,060,190 | 9,470 | 631 | 53 |
| 2018 | 2,443,550 | 8,885 | 1,198 | 99 |
| 2019 | 2,736,536 | 6,372 | 2,366 | 355 |
| 2020 | 2,642,901 | 4,309 | 2,184 | 340 |
| 2021 | 5,323,252 | 5,658 | 2,187 | 609 |
| 2022 | 3,399,126 | 3,307 | 1,939 | 612 |
| Notifier type |  |  |  |  |
| Physician | 8,679,185 | 47,597 | 6,928 | 1593 |
| Pharmacist | 2,429,733 | 7,093 | 785 | 109 |
| Other Health Professional | 4,068,553 | 14,492 | 1,262 | 270 |
| Lawyer | 504,000 | 102 | 4 | NA |
| Consumer or other non-health professional | 9,262,132 | 10,177 | 1,495 | 80 |
| NA | 7,577,380 | 5,490 | 121 | 40 |

**Supplementary Table 3**. Reporting odds ratios for cardiovascular toxicity among reports for males

| **Adverse event** | **Drug** | **Number of reports** | **ROR** | **95% CI min of ROR** | **IC** | **95% CI min of IC** |
| --- | --- | --- | --- | --- | --- | --- |
| Hypertension | Bevacizumab | 1,193 | 3.66 | 3.45 | 1.82 | 1.72 |
| Hypertension | Atezolizumab | 31 | 0.51 | 0.36 | -0.95 | -1.55 |
| Hypertension | Atezolizumab and bevacizumab | 53 | 4.41 | 3.35 | 2.04 | 1.59 |
| Arterial embolism and thrombosis | Bevacizumab | 682 | 2.07 | 1.92 | 1.03 | 0.9 |
| Arterial embolism and thrombosis | Atezolizumab | 34 | 0.57 | 0.4 | -0.8 | -1.37 |
| Arterial embolism and thrombosis | Atezolizumab and bevacizumab | 11 | 0.89 | 0.49 | -0.16 | -1.19 |
| Myocardial infarction | Bevacizumab | 339 | 1.23 | 1.1 | 0.29 | 0.11 |
| Myocardial infarction | Atezolizumab | 32 | 0.65 | 0.46 | -0.62 | -1.21 |
| Myocardial infarction | Atezolizumab and bevacizumab | 7 | 0.68 | 0.32 | -0.52 | -1.82 |
| Central nervous system ischemia | Bevacizumab | 500 | 2.35 | 2.16 | 1.22 | 1.07 |
| Central nervous system ischemia | Atezolizumab | 16 | 0.42 | 0.25 | -1.24 | -2.08 |
| Central nervous system ischemia | Atezolizumab and bevacizumab | 8 | 1.01 | 0.5 | 0.01 | -1.2 |
| Endocardial disorders | Bevacizumab | 5 | 0.94 | 0.39 | -0.09 | -1.65 |
| Endocardial disorders | Atezolizumab | 2 | 2.1 | 0.52 | 0.78 | -1.81 |
| Endocardial disorders | Atezolizumab and bevacizumab | 1 | 5.07 | 0.71 | 1.1 | -2.68 |
| Supraventricular tachyarrhythmias | Bevacizumab | 230 | 2 | 1.75 | 0.98 | 0.77 |
| Supraventricular tachyarrhythmias | Atezolizumab | 66 | 3.22 | 2.53 | 1.65 | 1.24 |
| Supraventricular tachyarrhythmias | Atezolizumab and bevacizumab | 7 | 1.64 | 0.78 | 0.65 | -0.65 |
| Ventricular tachyarrhythmias | Bevacizumab | 41 | 1.15 | 0.85 | 0.2 | -0.32 |
| Ventricular tachyarrhythmias | Atezolizumab | 3 | 0.47 | 0.15 | -0.97 | -3.04 |
| Ventricular tachyarrhythmias | Atezolizumab and bevacizumab | 0 | 0 | 0 | -1.86 | -12.19 |
| Heart failure | Bevacizumab | 222 | 1.29 | 1.13 | 0.36 | 0.14 |
| Heart failure | Atezolizumab | 33 | 1.07 | 0.76 | 0.09 | -0.49 |
| Heart failure | Atezolizumab and bevacizumab | 9 | 1.41 | 0.73 | 0.46 | -0.68 |
| Shock | Bevacizumab | 256 | 1.33 | 1.18 | 0.41 | 0.2 |
| Shock | Atezolizumab | 34 | 0.99 | 0.71 | -0.02 | -0.59 |
| Shock | Atezolizumab and bevacizumab | 3 | 0.42 | 0.14 | -1.12 | -3.19 |
| Myocarditis | Bevacizumab | 6 | 0.1 | 0.04 | -3.27 | -4.69 |
| Myocarditis | Atezolizumab | 32 | 2.89 | 2.04 | 1.49 | 0.9 |
| Myocarditis | Atezolizumab and bevacizumab | 10 | 4.38 | 2.35 | 1.91 | 0.83 |
| Pericarditis | Bevacizumab | 52 | 0.88 | 0.67 | -0.18 | -0.64 |
| Pericarditis | Atezolizumab | 27 | 2.58 | 1.77 | 1.32 | 0.68 |
| Pericarditis | Atezolizumab and bevacizumab | 3 | 1.38 | 0.44 | 0.39 | -1.68 |
| Hemorrhage-related clinical events | Bevacizumab | 2,128 | 2.4 | 2.3 | 1.2 | 1.13 |
| Hemorrhage-related clinical events | Atezolizumab | 123 | 0.74 | 0.62 | -0.43 | -0.72 |
| Hemorrhage-related clinical events | Atezolizumab and bevacizumab | 52 | 1.54 | 1.17 | 0.59 | 0.13 |
| Bleeding-related laboratory abnormalities | Bevacizumab | 24 | 1.24 | 0.83 | 0.3 | -0.38 |
| Bleeding-related laboratory abnormalities | Atezolizumab | 0 | 0 | 0 | -2.99 | -13.31 |
| Bleeding-related laboratory abnormalities | Atezolizumab and bevacizumab | 0 | 0 | 0 | -1.28 | -11.61 |
| Cerebral hemorrhage | Bevacizumab | 405 | 1.76 | 1.6 | 0.81 | 0.64 |
| Cerebral hemorrhage | Atezolizumab | 22 | 0.53 | 0.35 | -0.89 | -1.61 |
| Cerebral hemorrhage | Atezolizumab and bevacizumab | 7 | 0.82 | 0.39 | -0.27 | -1.57 |
| Pulmonary hypertension | Bevacizumab | 50 | 1.79 | 1.36 | 0.83 | 0.36 |
| Pulmonary hypertension | Atezolizumab | 2 | 0.4 | 0.1 | -1.13 | -3.73 |
| Pulmonary hypertension | Atezolizumab and bevacizumab | 0 | 0 | 0 | -1.62 | -11.94 |
| Vasculitis | Bevacizumab | 33 | 0.85 | 0.6 | -0.23 | -0.81 |
| Vasculitis | Atezolizumab | 10 | 1.44 | 0.78 | 0.5 | -0.58 |
| Vasculitis | Atezolizumab and bevacizumab | 1 | 0.7 | 0.1 | -0.37 | -4.15 |
| Temporal arteritis/polymyalgia rheumatica | Bevacizumab | 3 | 0.47 | 0.15 | -0.97 | -3.04 |
| Temporal arteritis/polymyalgia rheumatica | Atezolizumab | 5 | 4.42 | 1.84 | 1.75 | 0.19 |
| Temporal arteritis/polymyalgia rheumatica | Atezolizumab and bevacizumab | 0 | 0 | 0 | -0.56 | -10.88 |
| Venous embolism and thrombosis | Bevacizumab | 1,137 | 7.54 | 7.1 | 2.84 | 2.74 |
| Venous embolism and thrombosis | Atezolizumab | 66 | 2.35 | 1.85 | 1.21 | 0.8 |
| Venous embolism and thrombosis | Atezolizumab and bevacizumab | 20 | 3.46 | 2.22 | 1.69 | 0.94 |
| Bradyarrhythmias | Bevacizumab | 42 | 0.86 | 0.64 | -0.21 | -0.72 |
| Bradyarrhythmias | Atezolizumab | 8 | 0.92 | 0.46 | -0.11 | -1.32 |
| Bradyarrhythmias | Atezolizumab and bevacizumab | 2 | 1.11 | 0.28 | 0.12 | -2.47 |
| Cardiomyopathy | Bevacizumab | 89 | 2.17 | 1.76 | 1.1 | 0.75 |
| Cardiomyopathy | Atezolizumab | 6 | 0.82 | 0.37 | -0.27 | -1.69 |
| Cardiomyopathy | Atezolizumab and bevacizumab | 6 | 3.96 | 1.77 | 1.68 | 0.27 |
| Respiratory failure | Bevacizumab | 243 | 1.39 | 1.22 | 0.47 | 0.25 |
| Respiratory failure | Atezolizumab | 69 | 2.22 | 1.75 | 1.13 | 0.73 |
| Respiratory failure | Atezolizumab and bevacizumab | 9 | 1.39 | 0.72 | 0.44 | -0.7 |

CI, confidence interval; ROR, reporting odds ratios; IC, information components

**Supplementary Table 4**. Reporting odds ratios for cardiovascular toxicity among reports for females

| **Adverse event** | **Drug** | **Number of reports** | **ROR** | **95% CI min of ROR** | **IC** | **95% CI min of IC** |
| --- | --- | --- | --- | --- | --- | --- |
| Hypertension | Bevacizumab | 2,924 | 5.86 | 5.64 | 2.45 | 2.39 |
| Hypertension | Atezolizumab | 42 | 0.81 | 0.6 | -0.29 | -0.8 |
| Hypertension | Atezolizumab and bevacizumab | 52 | 5.92 | 4.47 | 2.42 | 1.96 |
| Arterial embolism and thrombosis | Bevacizumab | 720 | 2.86 | 2.66 | 1.49 | 1.37 |
| Arterial embolism and thrombosis | Atezolizumab | 30 | 1.22 | 0.85 | 0.28 | -0.33 |
| Arterial embolism and thrombosis | Atezolizumab and bevacizumab | 14 | 3.18 | 1.87 | 1.55 | 0.65 |
| Myocardial infarction | Bevacizumab | 327 | 1.87 | 1.68 | 0.89 | 0.71 |
| Myocardial infarction | Atezolizumab | 25 | 1.48 | 1 | 0.55 | -0.12 |
| Myocardial infarction | Atezolizumab and bevacizumab | 4 | 1.31 | 0.49 | 0.34 | -1.43 |
| Central nervous system ischemia | Bevacizumab | 614 | 2.54 | 2.34 | 1.32 | 1.19 |
| Central nervous system ischemia | Atezolizumab | 27 | 1.14 | 0.78 | 0.19 | -0.45 |
| Central nervous system ischemia | Atezolizumab and bevacizumab | 9 | 2.12 | 1.1 | 0.99 | -0.15 |
| Endocardial disorders | Bevacizumab | 7 | 1.61 | 0.77 | 0.63 | -0.68 |
| Endocardial disorders | Atezolizumab | 0 | 0 | 0 | -0.88 | -11.2 |
| Endocardial disorders | Atezolizumab and bevacizumab | 0 | 0 | 0 | -0.2 | -10.53 |
| Supraventricular tachyarrhythmias | Bevacizumab | 241 | 2.17 | 1.91 | 1.1 | 0.89 |
| Supraventricular tachyarrhythmias | Atezolizumab | 24 | 2.23 | 1.5 | 1.12 | 0.44 |
| Supraventricular tachyarrhythmias | Atezolizumab and bevacizumab | 5 | 2.57 | 1.07 | 1.17 | -0.4 |
| Ventricular tachyarrhythmias | Bevacizumab | 57 | 1.76 | 1.36 | 0.8 | 0.36 |
| Ventricular tachyarrhythmias | Atezolizumab | 4 | 1.28 | 0.48 | 0.31 | -1.45 |
| Ventricular tachyarrhythmias | Atezolizumab and bevacizumab | 0 | 0 | 0 | -1.09 | -11.42 |
| Heart failure | Bevacizumab | 524 | 3.06 | 2.81 | 1.59 | 1.45 |
| Heart failure | Atezolizumab | 36 | 2.17 | 1.56 | 1.09 | 0.53 |
| Heart failure | Atezolizumab and bevacizumab | 9 | 3 | 1.56 | 1.43 | 0.29 |
| Shock | Bevacizumab | 244 | 1.4 | 1.24 | 0.48 | 0.27 |
| Shock | Atezolizumab | 14 | 0.83 | 0.49 | -0.25 | -1.16 |
| Shock | Atezolizumab and bevacizumab | 2 | 0.66 | 0.16 | -0.5 | -3.09 |
| Myocarditis | Bevacizumab | 6 | 0.22 | 0.1 | -2.08 | -3.5 |
| Myocarditis | Atezolizumab | 27 | 10.43 | 7.14 | 3.14 | 2.5 |
| Myocarditis | Atezolizumab and bevacizumab | 2 | 4.24 | 1.06 | 1.36 | -1.23 |
| Pericarditis | Bevacizumab | 103 | 1.94 | 1.6 | 0.95 | 0.62 |
| Pericarditis | Atezolizumab | 22 | 4.31 | 2.83 | 2 | 1.28 |
| Pericarditis | Atezolizumab and bevacizumab | 1 | 1.08 | 0.15 | 0.07 | -3.71 |
| Hemorrhage-related clinical events | Bevacizumab | 2,703 | 1.92 | 1.85 | 0.89 | 0.83 |
| Hemorrhage-related clinical events | Atezolizumab | 67 | 0.47 | 0.37 | -1.06 | -1.46 |
| Hemorrhage-related clinical events | Atezolizumab and bevacizumab | 31 | 1.23 | 0.86 | 0.28 | -0.32 |
| Bleeding-related laboratory abnormalities | Bevacizumab | 35 | 1.84 | 1.32 | 0.86 | 0.3 |
| Bleeding-related laboratory abnormalities | Atezolizumab | 1 | 0.55 | 0.08 | -0.64 | -4.42 |
| Bleeding-related laboratory abnormalities | Atezolizumab and bevacizumab | 1 | 3.02 | 0.42 | 0.85 | -2.93 |
| Cerebral hemorrhage | Bevacizumab | 542 | 2.36 | 2.17 | 1.22 | 1.08 |
| Cerebral hemorrhage | Atezolizumab | 25 | 1.12 | 0.75 | 0.16 | -0.51 |
| Cerebral hemorrhage | Atezolizumab and bevacizumab | 5 | 1.24 | 0.51 | 0.27 | -1.29 |
| Pulmonary hypertension | Bevacizumab | 93 | 1.86 | 1.51 | 0.88 | 0.54 |
| Pulmonary hypertension | Atezolizumab | 4 | 0.83 | 0.31 | -0.25 | -2.01 |
| Pulmonary hypertension | Atezolizumab and bevacizumab | 0 | 0 | 0 | -1.46 | -11.78 |
| Vasculitis | Bevacizumab | 57 | 1.18 | 0.91 | 0.24 | -0.2 |
| Vasculitis | Atezolizumab | 5 | 1.07 | 0.45 | 0.09 | -1.47 |
| Vasculitis | Atezolizumab and bevacizumab | 1 | 1.19 | 0.17 | 0.16 | -3.62 |
| Temporal arteritis/polymyalgia rheumatica | Bevacizumab | 7 | 0.83 | 0.4 | -0.25 | -1.55 |
| Temporal arteritis/polymyalgia rheumatica | Atezolizumab | 1 | 1.23 | 0.17 | 0.19 | -3.59 |
| Temporal arteritis/polymyalgia rheumatica | Atezolizumab and bevacizumab | 0 | 0 | 0 | -0.37 | -10.69 |
| Venous embolism and thrombosis | Bevacizumab | 1,222 | 5.41 | 5.11 | 2.39 | 2.29 |
| Venous embolism and thrombosis | Atezolizumab | 41 | 1.83 | 1.35 | 0.85 | 0.33 |
| Venous embolism and thrombosis | Atezolizumab and bevacizumab | 14 | 3.49 | 2.05 | 1.67 | 0.76 |
| Bradyarrhythmias | Bevacizumab | 65 | 1.36 | 1.07 | 0.44 | 0.03 |
| Bradyarrhythmias | Atezolizumab | 3 | 0.65 | 0.21 | -0.55 | -2.62 |
| Bradyarrhythmias | Atezolizumab and bevacizumab | 2 | 2.4 | 0.6 | 0.91 | -1.69 |
| Cardiomyopathy | Bevacizumab | 225 | 6.18 | 5.41 | 2.59 | 2.37 |
| Cardiomyopathy | Atezolizumab | 22 | 6.2 | 4.07 | 2.47 | 1.75 |
| Cardiomyopathy | Atezolizumab and bevacizumab | 3 | 4.66 | 1.5 | 1.61 | -0.46 |
| Respiratory failure | Bevacizumab | 290 | 1.69 | 1.5 | 0.75 | 0.55 |
| Respiratory failure | Atezolizumab | 40 | 2.42 | 1.77 | 1.24 | 0.71 |
| Respiratory failure | Atezolizumab and bevacizumab | 10 | 3.35 | 1.79 | 1.58 | 0.5 |

CI, confidence interval; ROR, reporting odds ratios; IC, information components

**Supplementary Table 5**. Reporting odds ratio for cardiovascular toxicity among reports for young individuals

| **Adverse event** | **Drug** | **Number of reports** | **ROR** | **95% CI min of ROR** | **IC** | **95% CI min of IC** |
| --- | --- | --- | --- | --- | --- | --- |
| Hypertension | Bevacizumab | 2,803 | 5.68 | 5.47 | 2.42 | 2.36 |
| Hypertension | Atezolizumab | 53 | 0.75 | 0.57 | -0.41 | -0.87 |
| Hypertension | Atezolizumab and bevacizumab | 79 | 5.75 | 4.58 | 2.41 | 2.04 |
| Arterial embolism and thrombosis | Bevacizumab | 845 | 2.69 | 2.51 | 1.4 | 1.29 |
| Arterial embolism and thrombosis | Atezolizumab | 37 | 0.86 | 0.62 | -0.22 | -0.76 |
| Arterial embolism and thrombosis | Atezolizumab and bevacizumab | 14 | 1.6 | 0.94 | 0.64 | -0.26 |
| Myocardial infarction | Bevacizumab | 390 | 1.56 | 1.41 | 0.64 | 0.47 |
| Myocardial infarction | Atezolizumab | 32 | 0.95 | 0.67 | -0.08 | -0.67 |
| Myocardial infarction | Atezolizumab and bevacizumab | 5 | 0.72 | 0.3 | -0.43 | -1.99 |
| Central nervous system ischemia | Bevacizumab | 589 | 2.7 | 2.49 | 1.41 | 1.28 |
| Central nervous system ischemia | Atezolizumab | 27 | 0.91 | 0.62 | -0.14 | -0.78 |
| Central nervous system ischemia | Atezolizumab and bevacizumab | 12 | 1.98 | 1.12 | 0.92 | -0.06 |
| Endocardial disorders | Bevacizumab | 10 | 1.75 | 0.94 | 0.75 | -0.33 |
| Endocardial disorders | Atezolizumab | 2 | 2.59 | 0.65 | 0.97 | -1.62 |
| Endocardial disorders | Atezolizumab and bevacizumab | 0 | 0 | 0 | -0.4 | -10.72 |
| Supraventricular tachyarrhythmias | Bevacizumab | 259 | 2.22 | 1.96 | 1.14 | 0.93 |
| Supraventricular tachyarrhythmias | Atezolizumab | 61 | 3.88 | 3.02 | 1.91 | 1.49 |
| Supraventricular tachyarrhythmias | Atezolizumab and bevacizumab | 8 | 2.48 | 1.24 | 1.18 | -0.03 |
| Ventricular tachyarrhythmias | Bevacizumab | 71 | 1.65 | 1.31 | 0.71 | 0.32 |
| Ventricular tachyarrhythmias | Atezolizumab | 7 | 1.2 | 0.57 | 0.25 | -1.06 |
| Ventricular tachyarrhythmias | Atezolizumab and bevacizumab | 0 | 0 | 0 | -1.76 | -12.08 |
| Heart failure | Bevacizumab | 455 | 2.54 | 2.32 | 1.33 | 1.17 |
| Heart failure | Atezolizumab | 44 | 1.81 | 1.35 | 0.84 | 0.34 |
| Heart failure | Atezolizumab and bevacizumab | 15 | 3.03 | 1.82 | 1.5 | 0.63 |
| Shock | Bevacizumab | 331 | 1.35 | 1.21 | 0.43 | 0.25 |
| Shock | Atezolizumab | 29 | 0.87 | 0.61 | -0.19 | -0.81 |
| Shock | Atezolizumab and bevacizumab | 5 | 0.74 | 0.31 | -0.41 | -1.97 |
| Myocarditis | Bevacizumab | 8 | 0.14 | 0.07 | -2.76 | -3.97 |
| Myocarditis | Atezolizumab | 41 | 5.37 | 3.95 | 2.34 | 1.82 |
| Myocarditis | Atezolizumab and bevacizumab | 8 | 5.12 | 2.56 | 2.04 | 0.82 |
| Pericarditis | Bevacizumab | 106 | 1.55 | 1.28 | 0.62 | 0.3 |
| Pericarditis | Atezolizumab | 38 | 4.13 | 3 | 1.98 | 1.44 |
| Pericarditis | Atezolizumab and bevacizumab | 3 | 1.59 | 0.51 | 0.55 | -1.52 |
| Hemorrhage-related clinical events | Bevacizumab | 2,911 | 2.24 | 2.16 | 1.11 | 1.05 |
| Hemorrhage-related clinical events | Atezolizumab | 116 | 0.63 | 0.52 | -0.65 | -0.95 |
| Hemorrhage-related clinical events | Atezolizumab and bevacizumab | 55 | 1.5 | 1.14 | 0.56 | 0.11 |
| Bleeding-related laboratory abnormalities | Bevacizumab | 25 | 1.25 | 0.85 | 0.32 | -0.35 |
| Bleeding-related laboratory abnormalities | Atezolizumab | 0 | 0 | 0 | -2.68 | -13 |
| Bleeding-related laboratory abnormalities | Atezolizumab and bevacizumab | 1 | 1.82 | 0.26 | 0.51 | -3.27 |
| Cerebral hemorrhage | Bevacizumab | 512 | 2.39 | 2.19 | 1.24 | 1.1 |
| Cerebral hemorrhage | Atezolizumab | 25 | 0.86 | 0.58 | -0.22 | -0.89 |
| Cerebral hemorrhage | Atezolizumab and bevacizumab | 7 | 1.18 | 0.56 | 0.22 | -1.09 |
| Pulmonary hypertension | Bevacizumab | 97 | 2.12 | 1.73 | 1.07 | 0.73 |
| Pulmonary hypertension | Atezolizumab | 5 | 0.81 | 0.33 | -0.29 | -1.85 |
| Pulmonary hypertension | Atezolizumab and bevacizumab | 0 | 0 | 0 | -1.82 | -12.15 |
| Vasculitis | Bevacizumab | 51 | 0.91 | 0.69 | -0.13 | -0.59 |
| Vasculitis | Atezolizumab | 8 | 1.06 | 0.53 | 0.08 | -1.13 |
| Vasculitis | Atezolizumab and bevacizumab | 2 | 1.3 | 0.32 | 0.29 | -2.3 |
| Temporal arteritis/polymyalgia rheumatica | Bevacizumab | 4 | 0.57 | 0.22 | -0.73 | -2.5 |
| Temporal arteritis/polymyalgia rheumatica | Atezolizumab | 3 | 3.19 | 1.03 | 1.28 | -0.79 |
| Temporal arteritis/polymyalgia rheumatica | Atezolizumab and bevacizumab | 0 | 0 | 0 | -0.47 | -10.79 |
| Venous embolism and thrombosis | Bevacizumab | 1,530 | 6.87 | 6.53 | 2.72 | 2.64 |
| Venous embolism and thrombosis | Atezolizumab | 79 | 2.55 | 2.04 | 1.32 | 0.95 |
| Venous embolism and thrombosis | Atezolizumab and bevacizumab | 22 | 3.48 | 2.28 | 1.71 | 0.99 |
| Bradyarrhythmias | Bevacizumab | 66 | 1.12 | 0.88 | 0.17 | -0.24 |
| Bradyarrhythmias | Atezolizumab | 7 | 0.88 | 0.42 | -0.17 | -1.47 |
| Bradyarrhythmias | Atezolizumab and bevacizumab | 2 | 1.23 | 0.31 | 0.24 | -2.36 |
| Cardiomyopathy | Bevacizumab | 203 | 4.19 | 3.65 | 2.04 | 1.81 |
| Cardiomyopathy | Atezolizumab | 23 | 3.49 | 2.32 | 1.73 | 1.03 |
| Cardiomyopathy | Atezolizumab and bevacizumab | 8 | 5.95 | 2.97 | 2.2 | 0.99 |
| Respiratory failure | Bevacizumab | 343 | 1.49 | 1.34 | 0.56 | 0.39 |
| Respiratory failure | Atezolizumab | 77 | 2.48 | 1.98 | 1.29 | 0.91 |
| Respiratory failure | Atezolizumab and bevacizumab | 13 | 2.04 | 1.18 | 0.97 | 0.03 |

CI, confidence interval; ROR, reporting odds ratios; IC, information components

**Supplementary Table 6**. Reporting odds ratios for cardiovascular toxicity among reports for older individuals

| **Adverse event** | **Drug** | **Number of reports** | **ROR** | **95% CI min of ROR** | **IC** | **95% CI min of IC** |
| --- | --- | --- | --- | --- | --- | --- |
| Hypertension | Bevacizumab | 448 | 4.19 | 3.81 | 1.98 | 1.83 |
| Hypertension | Atezolizumab | 11 | 0.51 | 0.28 | -0.92 | -1.95 |
| Hypertension | Atezolizumab and bevacizumab | 15 | 3.52 | 2.1 | 1.66 | 0.79 |
| Arterial embolism and thrombosis | Bevacizumab | 223 | 2.6 | 2.27 | 1.34 | 1.12 |
| Arterial embolism and thrombosis | Atezolizumab | 15 | 0.9 | 0.54 | -0.14 | -1.01 |
| Arterial embolism and thrombosis | Atezolizumab and bevacizumab | 8 | 2.37 | 1.17 | 1.11 | -0.1 |
| Myocardial infarction | Bevacizumab | 107 | 1.79 | 1.48 | 0.82 | 0.5 |
| Myocardial infarction | Atezolizumab | 14 | 1.23 | 0.73 | 0.28 | -0.62 |
| Myocardial infarction | Atezolizumab and bevacizumab | 3 | 1.27 | 0.41 | 0.29 | -1.78 |
| Central nervous system ischemia | Bevacizumab | 202 | 2.28 | 1.98 | 1.16 | 0.92 |
| Central nervous system ischemia | Atezolizumab | 11 | 0.64 | 0.35 | -0.61 | -1.64 |
| Central nervous system ischemia | Atezolizumab and bevacizumab | 4 | 1.13 | 0.42 | 0.16 | -1.61 |
| Endocardial disorders | Bevacizumab | 1 | 0.9 | 0.13 | -0.11 | -3.89 |
| Endocardial disorders | Atezolizumab | 0 | 0 | 0 | -0.51 | -10.83 |
| Endocardial disorders | Atezolizumab and bevacizumab | 1 | 22.99 | 3.22 | 1.46 | -2.32 |
| Supraventricular tachyarrhythmias | Bevacizumab | 79 | 1.63 | 1.3 | 0.69 | 0.31 |
| Supraventricular tachyarrhythmias | Atezolizumab | 28 | 3.07 | 2.11 | 1.55 | 0.92 |
| Supraventricular tachyarrhythmias | Atezolizumab and bevacizumab | 3 | 1.57 | 0.5 | 0.54 | -1.53 |
| Ventricular tachyarrhythmias | Bevacizumab | 7 | 0.64 | 0.31 | -0.6 | -1.9 |
| Ventricular tachyarrhythmias | Atezolizumab | 0 | 0 | 0 | -2.35 | -12.67 |
| Ventricular tachyarrhythmias | Atezolizumab and bevacizumab | 0 | 0 | 0 | -0.89 | -11.21 |
| Heart failure | Bevacizumab | 90 | 1.24 | 1.01 | 0.3 | -0.05 |
| Heart failure | Atezolizumab | 15 | 1.09 | 0.65 | 0.12 | -0.75 |
| Heart failure | Atezolizumab and bevacizumab | 2 | 0.7 | 0.17 | -0.42 | -3.02 |
| Shock | Bevacizumab | 52 | 0.92 | 0.7 | -0.12 | -0.58 |
| Shock | Atezolizumab | 13 | 1.22 | 0.7 | 0.27 | -0.67 |
| Shock | Atezolizumab and bevacizumab | 0 | 0 | 0 | -2.44 | -12.77 |
| Myocarditis | Bevacizumab | 2 | 0.81 | 0.2 | -0.25 | -2.85 |
| Myocarditis | Atezolizumab | 11 | 23.93 | 13.18 | 3.57 | 2.55 |
| Myocarditis | Atezolizumab and bevacizumab | 3 | 31.34 | 10.03 | 2.55 | 0.48 |
| Pericarditis | Bevacizumab | 12 | 1.45 | 0.82 | 0.5 | -0.47 |
| Pericarditis | Atezolizumab | 4 | 2.55 | 0.96 | 1.12 | -0.65 |
| Pericarditis | Atezolizumab and bevacizumab | 0 | 0 | 0 | -0.72 | -11.05 |
| Hemorrhage-related clinical events | Bevacizumab | 555 | 1.23 | 1.13 | 0.28 | 0.14 |
| Hemorrhage-related clinical events | Atezolizumab | 41 | 0.46 | 0.34 | -1.06 | -1.58 |
| Hemorrhage-related clinical events | Atezolizumab and bevacizumab | 17 | 0.95 | 0.58 | -0.07 | -0.89 |
| Bleeding-related laboratory abnormalities | Bevacizumab | 8 | 1.22 | 0.61 | 0.26 | -0.95 |
| Bleeding-related laboratory abnormalities | Atezolizumab | 0 | 0 | 0 | -1.8 | -12.13 |
| Bleeding-related laboratory abnormalities | Atezolizumab and bevacizumab | 0 | 0 | 0 | -0.6 | -10.92 |
| Cerebral hemorrhage | Bevacizumab | 149 | 1.31 | 1.11 | 0.38 | 0.11 |
| Cerebral hemorrhage | Atezolizumab | 13 | 0.6 | 0.35 | -0.71 | -1.65 |
| Cerebral hemorrhage | Atezolizumab and bevacizumab | 4 | 0.89 | 0.33 | -0.14 | -1.91 |
| Pulmonary hypertension | Bevacizumab | 15 | 1.32 | 0.79 | 0.38 | -0.49 |
| Pulmonary hypertension | Atezolizumab | 1 | 0.46 | 0.07 | -0.82 | -4.61 |
| Pulmonary hypertension | Atezolizumab and bevacizumab | 0 | 0 | 0 | -0.92 | -11.24 |
| Vasculitis | Bevacizumab | 21 | 1.65 | 1.07 | 0.69 | -0.04 |
| Vasculitis | Atezolizumab | 3 | 1.24 | 0.4 | 0.26 | -1.81 |
| Vasculitis | Atezolizumab and bevacizumab | 0 | 0 | 0 | -1 | -11.32 |
| Temporal arteritis/polymyalgia rheumatica | Bevacizumab | 1 | 0.28 | 0.04 | -1.43 | -5.22 |
| Temporal arteritis/polymyalgia rheumatica | Atezolizumab | 3 | 4.48 | 1.44 | 1.58 | -0.49 |
| Temporal arteritis/polymyalgia rheumatica | Atezolizumab and bevacizumab | 0 | 0 | 0 | -0.35 | -10.68 |
| Venous embolism and thrombosis | Bevacizumab | 221 | 5.39 | 4.71 | 2.37 | 2.14 |
| Venous embolism and thrombosis | Atezolizumab | 20 | 2.51 | 1.62 | 1.26 | 0.51 |
| Venous embolism and thrombosis | Atezolizumab and bevacizumab | 6 | 3.66 | 1.63 | 1.59 | 0.17 |
| Bradyarrhythmias | Bevacizumab | 17 | 0.87 | 0.54 | -0.2 | -1.02 |
| Bradyarrhythmias | Atezolizumab | 4 | 1.08 | 0.4 | 0.1 | -1.67 |
| Bradyarrhythmias | Atezolizumab and bevacizumab | 1 | 1.3 | 0.18 | 0.24 | -3.54 |
| Cardiomyopathy | Bevacizumab | 21 | 2.73 | 1.78 | 1.38 | 0.65 |
| Cardiomyopathy | Atezolizumab | 0 | 0 | 0 | -1.97 | -12.3 |
| Cardiomyopathy | Atezolizumab and bevacizumab | 1 | 3.31 | 0.46 | 0.9 | -2.88 |
| Respiratory failure | Bevacizumab | 47 | 0.92 | 0.69 | -0.11 | -0.6 |
| Respiratory failure | Atezolizumab | 21 | 2.2 | 1.43 | 1.09 | 0.36 |
| Respiratory failure | Atezolizumab and bevacizumab | 5 | 2.54 | 1.05 | 1.14 | -0.42 |

CI, confidence interval; ROR, reporting odds ratios; IC, information components
